# Epigenetics and Autoimmune Diseases

**DOI:** 10.1155/2012/593720

**Published:** 2012-03-22

**Authors:** Paula Quintero-Ronderos, Gladis Montoya-Ortiz

**Affiliations:** Center for Autoimmune Diseases Research (CREA), School of Medicine and Health Sciences, Universidad del Rosario, Carrera 24 no. 63C-69 Bogotá, Colombia

## Abstract

Epigenetics is defined as the study of all inheritable and potentially reversible changes in genome function that do not alter the nucleotide sequence within the DNA. Epigenetic mechanisms such as DNA methylation, histone modification, nucleosome positioning, and microRNAs (miRNAs) are essential to carry out key functions in the regulation of gene expression. Therefore, the epigenetic mechanisms are a window to understanding the possible mechanisms involved in the pathogenesis of complex diseases such as autoimmune diseases. It is noteworthy that autoimmune diseases do not have the same epidemiology, pathology, or symptoms but do have a common origin that can be explained by the sharing of immunogenetic mechanisms. Currently, epigenetic research is looking for disruption in one or more epigenetic mechanisms to provide new insights into autoimmune diseases. The identification of cell-specific targets of epigenetic deregulation will serve us as clinical markers for diagnosis, disease progression, and therapy approaches.

## 1. Introduction

Epigenetics was defined by Conrad Waddington in the early 1940s as *the branch of biology that studies the causal interactions between genes and their products which bring the phenotype into being *[1]. Currently, epigenetics is defined as the *study of changes in gene function that are inheritable and that do not entail a change in DNA sequence *[2]. As has been mentioned before, all these mechanisms are inheritable thus the epigenetic markers have the ability to persist during development and potentially be transmitted from offspring to offspring. These mechanisms play an essential role in regulation of gene and miRNA expression, DNA-protein interactions, cell differentiation, embryogenesis, X-chromosome inactivation, and genomic imprinting [3].

One of the main functions of epigenetics is gene regulation. Gene regulation plays an important role in determining individual gene function and activity, the sets of genes which are functional in each specific cell type, cell type development and differentiation, and metabolic plasticity of the cell that allows it to adapt itself to environmental changes. However, it is important to note that epigenetics is not the only determinant of gene function. There are intrinsic components that are stable over time and are the same in each cell type. These intrinsic components, which include polymorphism and mutations, are among the mechanisms that affect gene expression. Also, the environment (virus, hormones, nutrition, and chemicals) influences epigenetics and thus, the intrinsic component altering gene function [4].

The interaction between environment and epigenetics is only one of the mechanisms by which a large range of different phenotypes arise from the same genotype such as in the case of monozygotic twins [5, 6]. Monozygotic twins have an identical DNA sequence, but studies have found some phenotypic differences that may be consequences of different exposures to environmental stressors. These exposures produce alterations in the DNA methylation pattern and histone modification. This condition may be one of the causes of the differences found in the concordance rate of autoimmune diseases between monozygotic twins (Table 1) [7–22].

**Figure 1:**
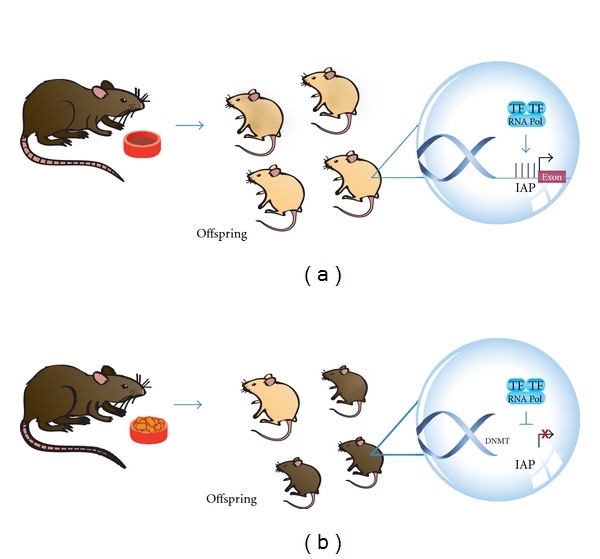
Epigenetic-environmental interaction. Offspring of pregnant *Agouti* rodents fed with food rich in methyl donors had a different color of coat (brown, (b)) due to an increased DNA methylation status in the viable yellow allele (*A* ^vy^ allele), in comparison to offspring of pregnant rodents fed a normal diet (yellow or mottle, (a)). Intracisternal A Particle (IAP), Transcription Factor (TF), RNA Polymerase (RNA Pol), Methylated Cytosine (M).

Another example of how epigenetics interact with the environment is in the study of pregnant *Agouti* rodents. In this study, researchers fed pregnant *Agouti* rodents with food rich in methyl donors such as folate, methionine, and choline. They found that, in comparison to offspring of pregnant rodents fed a normal diet (yellow or mottle, Figure 1(a)), the offspring of these rodents had a different color of coat (brown, Figure 1(b)) due to an increased DNA methylation status in the viable yellow allele (*A* ^vy^ allele). These authors demonstrated that the percentage of phenotypes with a darker brown coat rises as increasing levels of methyl supplement are added to the diet. The lack of a methyl supplement has important implications because it indicates a pattern of future obesity and insulin resistance. In other words, mice with yellow or mottle coats have altered metabolism and obesity. It also results in increased cancer susceptibility, adult diabetes, and twice the mortality seen in normal mice [58].

Other researchers showed that Dutch who were exposed prenatally to famine during the Dutch Famine of 1944 in World War II had less DNA methylation of the imprinted Insulin-like growth factor 2 (IGF-2) gene 6 decades later compared to their unexposed, same-sex siblings. Because of the lack of nutrients that these individuals suffered during prenatal life, there was a deficiency of methyl donors such as the amino acid methionine that causes the hypomethylation of the differentially methylated region (DMR) in the maternally imprinted IGF-2 gene in comparison to same sex siblings who were not exposed. Even though the IGF-2 gene plays a key role in human growth and development, there was no evidence with respect to the relationship between hypomethylation of IGF-2 and birth weight. Thus, this finding supports the hypothesis that early mammalian development is important for establishing and maintaining epigenetic markers [59, 60].

Many studies had been done of the cohort from the Dutch Winter of Famine in World War II. One of them looked for differences in birth weight between offspring of mothers who were exposed to famine in either early or late gestation. The authors found that individuals who were exposed to famine in early gestation had epigenetic differences but a normal birth weight. In contrast, individuals exposed to famine in late gestation had a low birth weight but did not have any epigenetic changes [58]. At the same time, other studies have demonstrated that those individuals exposed to famine during the gestational period have a higher risk of developing schizophrenia and dyslipidemia. One of these studies demonstrated that there are sex-specific differences in the pattern of atherogenic lipids at the age of 58. Women showed elevated serum concentrations of total cholesterol, low-density lipoprotein (LDL), and triglycerides in comparison to unexposed women [61, 62]. It was also found that exposed women had a wide range of body mass indexes and thus have a higher risk of obesity and developing chronic diseases [63–65]. Other studies have shown that individuals exposed to famine in early gestation, both males and females, have an increased risk of schizophrenia while individuals who were exposed in later gestation have a higher risk of developing affective disorders [66–68].

## 2. Epigenetic Mechanisms

There are different epigenetic mechanisms that regulate gene expression whether this is to activate or repress it: DNA methylation, histone modification, nucleosome positioning, and RNA interference (RNAi) (miRNAs and small interfering RNA (siRNAs)) [4]. It is important to mention that all these epigenetic mechanisms act together at the same time and not separately to regulate gene expression.

### 2.1. DNA Methylation

**Figure 2:**
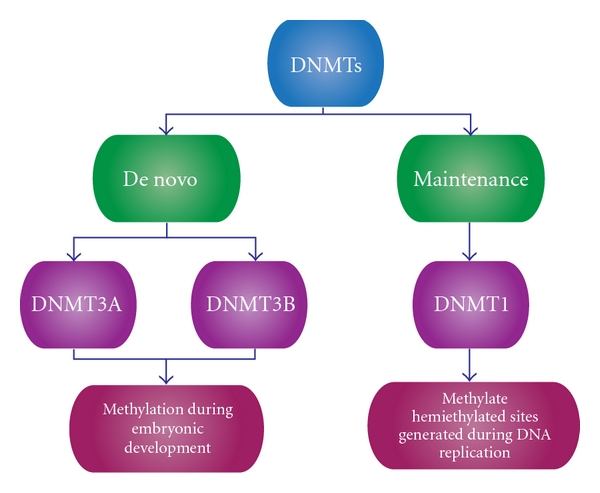
Classification of DNMTs. DNMTs can be classified into *de novo *and maintenance. *De novo *DNMTs are involved in methylation during embryonic development, and maintenance DNMTs are involved in methylation during DNA replication.

There are evidence that DNA methylation occurs in different regions of the genome and it has great importance in embryogenesis, cellular differentiation, and tissue-specific development. It is noteworthy that DNA methylation varies among tissues and cell type because it is a dynamic process involving methylation and demethylation events [69–71] and plays a role in normal regulatory functions. Therefore, a dysfunction of normal state DNA methylation would lead to disease. Methylation is mediated by the DNA methyltransferase (DNMTs) family which is responsible for donating a methyl group to the DNA 5-cystosine. This family of enzymes has 5 members: DNMT1, DNMT2, DNMT3a, DNMT3b, and DNMT3L. At the same time, DNMTs can be classified into *de Novo* and maintenance DNMTs (Figure 2) [4]. *De Novo* DNMTs are DNMT3a, and DNMT3b and they are responsible for methylation during embryonic development. DNMT1 is the maintenance DNMT which is responsible for methylate hemimethylated sites that are generated during DNA replication. DNMT2 acts on transfer RNA, and DNMT3L acts on embryogenesis [72].

The other mechanism that counteracts DNA methylation is demethylation. Demethylation can be passive or active [4]. The first is induced by inhibition of DNMTs activities such as in the case of several drugs that are used as therapeutic compounds to eliminate aberrant hypermethylation. Active demethylation, in turn, occurs in cell differentiation and has been found in the activation of immune cells [73]. This process depends on the action of cytosine deaminase, which, when it is activated, induces cytidine deaminase (AICDA) that deaminates 5-methylcytosine [74]. 

It is important to understand that when there is a methylation state, transcription will be repressed; in contrast, when there is an unmethylated state, transcription will be permitted. Transcription inhibition is achieved because methyl groups interfere with the binding of transcription factors that activate transcription from a specific gene. Many of these transcription factors recognize mainly CpG sequences, but when these sequences are methylated, they are unable to bind DNA. An additional mechanism of transcriptional repression involves proteins that are attracted to methylated CpG sequences. These protein families are part of the methyl-CpG-binding domain (MBD), and they recognize methylated sequences thus providing a further signal to alter chromatin structures by formation of a corepressor complex [75].

**Figure 3:**
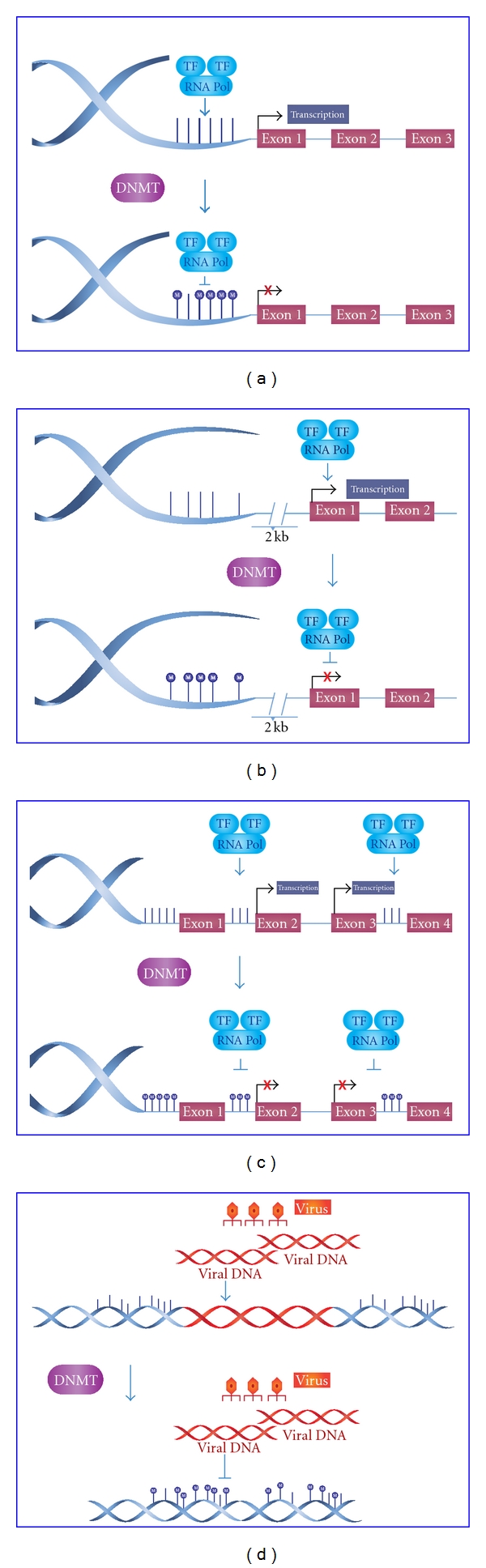
DNA methylation patterns. (a) In basal state, CpG Islands are unmethylated to allow the transcription, but when they are methylated at promoter regions of genes, the transcription will be inhibited. (b) At the same time, CpG island shores (located up to ~2 kb from CpG islands) have a methylation pattern that is similar to the CpG islands in that methylation is closely associated with transcriptional inactivation. (c) Gene bodies are methylated to prevent spurious transcription initiations. (d) Repetitive sequences which are hypermethylated to protect chromosomal integrity by preventing reactivation of endoparasitic sequences that cause chromosomal instability.

There are four possible DNA methylation patterns. The first methylation pattern and the most widely studied is the methylation of CpG islands at promoter regions of genes. These CpG islands are regions of more than 200 bases with a G + C content of at least 50%. Many human gene promoters (60%) are associated with CpG islands and their basal state must be unmethylated to allow transcription (Figure 3(a)) [75, 76]. The second pattern is DNA methylation of CpG island shores, which are regions of lower CpG density in close proximity (~2 kb) to CpG islands. This pattern is similar to the CpG island methylation pattern in which methylation is closely associated with transcriptional inactivation. It is important to note that most of the tissue-specific DNA methylation occurs in these regions (Figure 3(b)) [75, 77].

In contrast with both mechanisms mentioned above, the third pattern occurs in gene bodies which, in their basal state, are methylated to facilitate correct transcription thus preventing spurious transcription initiations (Figure 3(c)) [78]. In disease, gene bodies are demethylated to allow transcription to be initiated at incorrect sites. DNA methylation can also take place in CHG and CHH (H = A, C or T) sites in the human genome. This methylation has been found predominantly in stem cells and seems to be enriched in gene bodies directly correlated with gene expression. The last pattern to mention is hypermethylation of repetitive sequences that protect chromosomal integrity by preventing reactivation of endoparasitic sequences that cause chromosomal instability, translocations, and gene disruption (Figure 3(d)) [79].

### 2.2. Histone Modifications

Histones are conserved proteins that package and organize DNA. These proteins can be grouped in core histones (H2A, H2B, H3, and H4) and linker histones (H1 and H5). The linker histones bind to the DNA by sealing off the nucleosome at the location where DNA enters and leaves [80]. 

**Figure 4:**
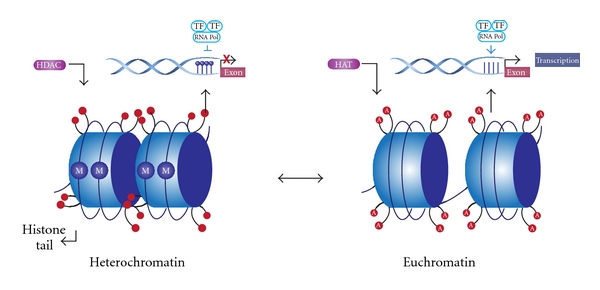
Histone modification. To form heterochromatin, histone deacetylation of histone tails caused by HDACs enzymes in association with DNA methylation (M) confers a dense configuration of DNA that prevents its transcription. In the euchromatin state, there is an acetylation of histone tails (A) by HATs enzymes in association with DNA demethylation to promote gene expression.

Histones suffer some posttranslational modifications such as lysine acetylation, and methylation, phosphorylation, ubiquitination, SUMOylation, and ADPribosylation. Histone modifications play an important role in transcriptional regulation, DNA repair, DNA replication and chromosome condensation [80, 81]. Of all these modifications, the one most widely studied is lysine acetylation. In this process, histones are acetylated and deacetylated on lysine residues in the N-terminal tail. These reactions are catalyzed by histone acetyltransferases (HATs), or histone deacetylases (HDACs) respectively [82, 83]. HATs promote gene expression by transferring an acetyl group to lysine and HDACs promote gene repression by removing an acetyl group from the lysine tail (Figure 4).

An example of how these modifications affect transcriptional regulation is the histone deacetylation with the association of 5′ methylcytosine in the DNA, which confers a heterochromatin configuration that makes DNA inaccessible to transcription factors. On the other hand, acetylation of histone tails such as lysine acetylation on histone 3 (H3K9) and DNA demethylation causes euchromatin configuration, which is accessible to transcription machinery [84]. It is important to mention that many posttranslational modifications can occur on the same histone tail and at same time produce the repression or the activation of gene expression [85]. For example, during cell cycle there is a regulatory relationship between methylation of H3K9 and phosphorylation of H3 serine 10 (H3S10). Phosphorylation of H3S10 is required for chromosomal condensation. During early prophase and anaphase, there are high quantities of H3S10 phosphorylation. In contrast, during late anaphase, dephosphorylation occurs and H3K9 methylation reemerges. Therefore, H3S10 phosphorylation blocked methylation of H3K9, which gave transcription factors access to DNA during mitosis. Also phosporylation preserves methylation patterns during cell division [86].

### 2.3. Nucleosome Positioning

Nucleosomes are a complex form of DNA packaged by histones. There are nucleosome positioning patterns that play an important role in transcriptional regulation. Depending on how close nucleosomes are to transcription start sites (TSSs), they may block the activators' and transcription factors' access to the DNA strand thus inhibiting elongation of the transcripts. Active gene promoters have a nucleosome-free region at the 5′ and 3′ untranslated region (UTR) to facilitate the assembly and disassembly of the transcription machinery [87]. For example, nucleosome displacements of as few as 30 bp at TSS have been implicated in changes in the activity of RNA polymerase II. When there is a loss of a nucleosome upstream from the TSS, transcription factors can bind to the TSSs and gene expression is achieved. In contrast, when there is an occlusion of the TSS by a nucleosome, transcription machinery does not bind to the TSSs and gene repression occurs. Interestingly, nucleosome positioning can influence DNA methylation because DNA methyltransferases preferentially target nucleosome-bound DNA [88].

### 2.4. microRNAs

miRNAs are RNAs that are 18–23 nucleotides in length and function as posttranscriptional regulators. They regulate mRNA translation by binding to complementary sequences that are cut or repressed. Many miRNAs are transcribed from intergenic regions or from introns of protein-coding genes and, sometimes, they are expressed at the same time that the protein gene is transcribed. Just a few miRNAs have been located in exons of protein-coding genes. Of all these miRNAs, the intergenic miRNAs are the only ones which have their own gene promoter and regulatory region [89].

**Figure 5:**
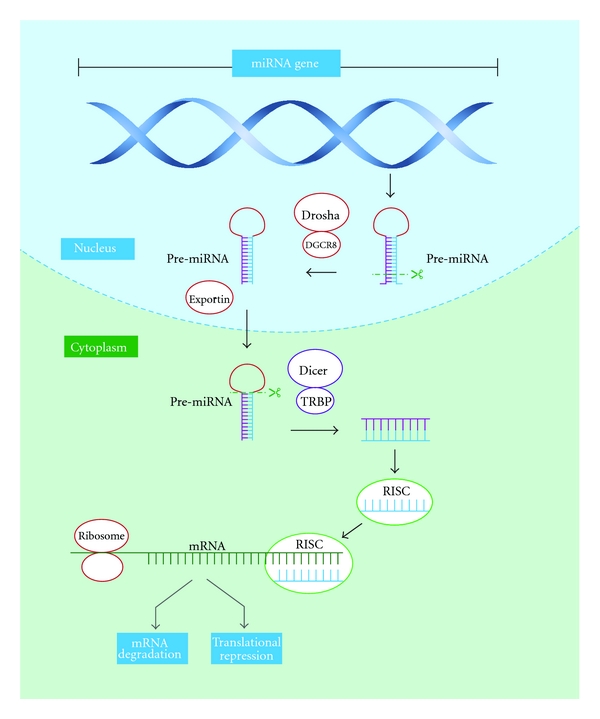
miRNA biogenesis. miRNA genes are transcribed by RNA Polymerase II in the nucleus to form a primary miRNA (pri-miRNA) that is, 100 to 1000 nucleotides in length. This pri-miRNA is recognized by nuclear enzymes Drosha, Pasha, or DGCR8 (in humans), which cleave about 11 nucleotides off of it to produce hairpin structures known as pre-miRNA, which are ~70 nucleotides in length. Once pre-miRNA hairpins are made, they are exported from the nucleus to the cytoplasm by the Exportin-5 enzyme. In the cytoplasm, the Dicer enzyme cleaves pre-miRNAs to form a duplex miRNA that is 18–23 nucleotides in length. Of these 2 strands, the one with lower stability in the 5′ end is the guide strand, and it will be associated with the RNA-induced silencing complex (RISC), where miRNAs interact with the mRNA targets. The RISC complex needs to interact with other proteins such as Argonaute (Ago) proteins and TRBP to function appropriately. The translational repression and target degradation of mRNAs can be achieved by the level of complementarity between miRNAs strand and the site in the 3′ UTR targets. If there is a complete complementation, there will be a cleavage of the mRNAs and it will produce the degradation. On the other hand, if there is an incomplete complementation, translation will be repressed by taking the transcripts into P bodies to keep them silenced.

The translational repression and target degradation of mRNAs is achieved by the level of complementarity between miRNA strands and the site in the 3′ UTR targets. If there is complete complementation, there will be cleavage of the mRNAs and this will produce degradation. On the other hand, if there is incomplete complementation, translation will be prevented by taking the transcripts into P bodies to keep them silenced using proteins that prevent translation or removal of the cap structure (Figure 5). Another mechanism by which miRNAs affect gene expression is by histone modification and DNA methylation of promoter sites. This mechanism occurs thanks to the RNA-induced transcriptional silencing (RITS) complex. This protein complex binds to miRNAs to perform posttranslational modification of histone tails such as methylation of H3K9 to form heterochromatin and to cause transcriptional repression [89, 90].

## 3. Epigenetics and Autoimmunity

Autoimmune diseases are a complex group of diseases that do not have the same epidemiology, pathology, or symptoms but do have a common origin [91]. All autoimmune diseases share immunogenetic mechanisms mediated in part by several pleiotropic genes. Many studies over the years have shown that these diseases are caused by alterations in many loci and genes in the human genome [92]. However, until recent years, epigenetic studies have focused on autoimmune diseases. Therefore, it is important to underline the fact that autoimmune diseases may be generated by several alterations in the same epigenetic mechanism. Also, it is essential to understand that epigenetics is not the only mechanism that may cause autoimmunity. In fact, there are intrinsic and extrinsic components (mutations, polymorphisms, and environmental factors) that predispose to autoimmunity.

### 3.1. DNA Methylation and Autoimmune Diseases

As was mentioned at the beginning of this paper, DNA methylation is the most widely studied mechanism in autoimmune diseases. Several studies done so far have found that some diseases such as systemic lupus erythematosus (SLE) and rheumatoid arthritis (RA) have global hypomethylation in the cells they target in promoter regions of DNA (Table 2). Studies of other autoimmune diseases in search of methylation patterns are just beginning.

#### 3.1.1. Systemic Lupus Erythematosus

SLE is a systemic multiorgan autoimmune disease characterized by autoantibody response to nuclear and/or cytoplasmic antigens. Several studies have shown that there is a global hypomethylation of promoter regions, which contain the genes that are overexpressed in the disease such as *ITGAL, CD40LG*, *PRF1, CD70, IFGNR2, MMP14, LCN2, *and in the ribosomal RNA gene promoter (18S and 28S) [23–27]. The DNA hypomethylation may also affect the chromatin structure of T-cells thus enhancing the overexpression of these genes. This gene overexpression will cause cell hyperactivity, perpetuation of the immune response and consequently, and perpetuation of inflammatory response [93–95].

An example of how hypomethylation alters gene expression in SLE is the hypomethylation of the E1B promoter of CD5 in resting B cells. CD5 is a protein found in B cells that serves to mitigate activating signals from the B cell receptor (BCR) so that B cells are only activated by strong stimuli and not by normal tissue proteins. CD5 has two isoforms: E1A that is expressed on the membrane and E1B that is retained in the cytoplasm. The hypomethylation of E1B promoters may be the consequence of a reduced expression of DNMT1. Therefore, there is an increase in the expression of this CD5 isoform that will cause impairment of cell receptor signaling, which will then promote autoimmunity [28].

Another example is in the Lupus like disease caused by procainamide and hydralazine. These two drugs are DNA methylation inhibitors. As a result, they produce hypomethylation of DNA [96]. Procainamide is a competitive inhibitor of DNMT1 [97]. In contrast, hydralazine inhibits T and B cell signal-regulated kinase pathways [98]. The kinase signaling pathway plays an important role in the regulation of methylation [99]. These two mechanisms produce a reduction in DNMTs that will enhance the genetic expression of adhesion molecules on lupus-drug-induced lymphocytes [100–102].

#### 3.1.2. Rheumatoid Arthritis

RA is a disease characterized by the progressive destruction of joints by invasive synovial fibroblasts. The RA synovial fibroblasts (RASFs) play a major role in the initiation and perpetuation of the disease [103]. They are the reason why several epigenetic studies of RA are focused on these synovial cells. Researchers have found a global hypomethylation of these cells, which could be responsible for the overexpression of inflammatory cytokines in synovial fluid [104, 105].

Some examples of hypomethylation in RA are in CpG islands upstream of an L1 open-reading frame and the Interleukin-6 (IL-6) promoter gene in monocytes. L1 is one of the major classes of repetitive elements that are spread throughout the genome. They are used as markers because they are methylated in normal synovial tissue. In synovial tissue from patients with RA, L1 is hypomethylated as a consequence of reduced expression of DNMTs. This reduction of methylation in inflammatory response promoter genes causes an overexpression of growth factors and receptors, adhesion molecules, and cytokines. In the end, they will cause irreversible phenotypic changes in synovial fibroblasts [29, 106].

The other example is the hypomethylation in CpG islands within the IL-6 promoter gene in monocytes. IL-6 is a proinflammatory cytokine that participates in B cell response. When this promoter is hypomethylated, there is an overexpression of IL-6 that will cause an overexpression of pro-inflammatory cytokines at the same time. This will be associated with a local hyperactivation of the inflammation circuit [30]. But there is evidence that we can also find a hypermethylation mechanism in monocytes such as in the case of the CpG islands within the promoter of death receptor 3 (DR-3). DR-3 is a protein that causes apoptosis and activation of transcription factor NF-kappa-B (NF-*κ*B). However, when there is a downregulation of this protein because of the hypermethylation of its promoter, the RA synovial cell will be resistant to apoptosis [31, 107, 108].

#### 3.1.3. Type 1 Diabetes (T1D)

T1D is a T-cell-mediated autoimmune disease that develops in genetically susceptible individuals and affects their endocrine pancreas. There are some mechanisms by which epigenetics may play an important role in T1D by modulating lymphocyte maturation and cytokine gene expression and by differentiation of subtype T helper cells ruled by epigenetic controls. In this autoimmune disease, in contrast to SLE and RA, there is a global hypermethylation activity caused by altered metabolism of homocysteine [109].

Glucose and insulin levels are determinants of methylation [32]. They alter homocysteine metabolism by increasing cell homocysteine production through its inhibition of trans-sulfuration [110, 111]. When there is an increase in the levels of homocysteine, methionine in cells will be catalyzed by DNMTs in S-adenosylmethionine. This will enhance DNMT activity that will subsequently lead to increased global DNA methylation. Also, an increase in maternal homocysteine during pregnancy as a result of a low protein diet can produce an altered methionine metabolism that will cause a decrease in islet mass and vascularity in the fetus with a subsequent glucose intolerance in adult life [112, 113].

#### 3.1.4. Multiple Sclerosis (MS)

MS is a chronic inflammatory disease characterized by myelin destruction followed by a progressive degree of neurodegeneration. Recent studies have shown that the promoter region of peptidyl arginine deiminase type II (PAD2) is hypomethylated [33]. PAD2 plays a key role in the citrullination process of myelin basic protein (MBP). This citrullination process has important biologic effects. It promotes protein autocleavage, which increases the probability of creating new epitopes and also modulates the immune response. In MS, an increase has been found in demethylase enzyme activity, which will cause hypomethylation of the PAD2 promoter region [114]. Because of this hypomethylation, there will be an overexpression of PAD2 that will increase the MBP citrullination process with a subsequent increase in the production of immunodominant peptides. These peptides will increase the autocleavage of MBP thereby causing irreversible changes in its biological properties, which will produce proteolytic digestion, myelin instability, and a chronic inflammation response [115–117].

#### 3.1.5. Systemic Sclerosis (SSc)

SSc is a rare condition of unknown etiology that is characterized by excessive collagen deposits on skin and other tissues with a progressive vasculopathy. In SSc, there is a hypermethylation of CpG islands in the Fli1 promoter, which is a transcription factor that inhibits collagen production. The reduced expression of Fli1 increases collagen synthesis, that will not be balanced by metalloproteinase activity. This promotes collagen accumulation and, subsequently, the tissue fibrosis that is a characteristic of the disease [34, 118].

### 3.2. Histone Modifications and Autoimmune Diseases

#### 3.2.1. Systemic Lupus Erythematosus

Histone modifications in SLE have been studied in murine models and in humans. These studies have found that, during apoptosis, histones can be modified to make them immunogenic. It is noteworthy that in the pathogenesis of SLE, antibodies are directed against components of the cell nucleus that are exposed at the cell surface during apoptosis [119, 120].

The nucleosomes, the primary inciting antigen in SLE, are released in patients with SLE as a result of a disturbed apoptosis or an insufficient clearance of apoptotic debris. During apoptosis, the nucleosome is modified, thereby creating more immunogenic epitopes. Subsequently, epitope spreading will lead to the formation of autoantibodies against unmodified chromatin components [35, 121]. Histone modifications such as histone 3 lysine 4 trimethylation (H3K4me3), histone 3 lysine 8 (H4K8) triacetylation, histone 3 lysine 27 trimethylation (H3K27me3), and histone 2B lysine 12 acetylation (H2BK12ac) will cause an increase in apoptotic nucleosomes (Table 2). These apoptotic nucleosomes will generate autoimmunogenicity that will cause activation of antigen-presenting cells and autoantibody production with a subsequent inflammatory response [36, 122].

There are other studies that have shown a global acetylation pattern of histone H3 and H4 in active SLE CD4+ T cells [123]. Also, monocytes, which are important in SLE renal disease, have been shown to have an altered acetylation pattern of histone H4 thus increasing the expression of interferon (IFN) genes that play a key role in SLE pathogenesis [124–126].

#### 3.2.2. Rheumatoid Arthritis

RA synovial tissue is characterized by an imbalance between HAT and HDAC activity. Cartilage destruction is thought to be mediated by matrix metalloproteinases (MMPs) and enzymes from the ADAMTS (a disintegrin and metalloproteinase domain with thrombospondin motifs) family. Many of these genes are regulated by modifications in the chromatin including acetylation of histones [127–129].

Many studies have shown that HDAC inhibitors inhibit cartilage degradation by blocking the induction of key MMPs by proinflammatory cytokines at both the mRNA and protein levels. Also, ADAMTs enzymes are inhibited at the mRNA level [37]. In fact, hyperacetylation of synovial cell histones induces p16 and p21 (cyclin-dependent kinase inhibitors that regulate cell cycle) expression with a subsequent decrease in Tumor Necrosis Factor-alpha (TNF-*α*) synthesis (Table 2). These mechanisms will inhibit joint swelling, synovial inflammation, and joint destruction in murine RA models [38, 129]. Also, the hyperacetylation of histones will downregulate HIF-1a (hypoxia inducible factor) and vascular endothelial growth factor (VEGF) to block angiogenesis in synovial cells [130].

It is noteworthy that HDAC inhibitors may, therefore, be new chondroprotective therapeutic agents in arthritis due to their ability to inhibit the expression of destructive metalloproteinases and ADAMTs in synovial tissue [131–133].

#### 3.2.3. Type 1 Diabetes

There are just a few epigenetic studies associated with histone modifications and the pathogenesis of T1D. Patients with T1D show a subset of genes with an increase in histone 3 lysine 9 dimethylation (H3K9me2) in lymphocytes. This subset of genes includes the *CLTA4,* which is a type 1 diabetes susceptibility gene and has increased methylation of H3K9 in its promoter region. Other genes that have altered H3K9me2 are transforming growth factor-beta (TGF-B), NF-*κ*B, p38 (mitogen-activated protein kinase), toll-like receptors (TLRs), and IL-6 (Table 2). The transcription factor NF-*κ*B is also upregulated by H3K4 methyltransferase thus causing an increase in inflammatory gene expression in diabetic mice. All these genes are associated with autoimmune and inflammation-related pathways [39, 134, 135].

Histone modifications are also among the mechanisms that cause cardiovascular complications in T1D patients. Chemical modification of the H3K4 and H3K9 has recently been found to be related to the gene expression conferred by hyperglycemia. Transient hyperglycemia promotes gene-activating epigenetic changes and signaling events critical in the development and progression of vascular complications. These epigenetic changes are H3K4 and H3K9 methylation in genes associated with vascular inflammation [40, 136, 137].

#### 3.2.4. Multiple Sclerosis

The oligodendrocyte identity is modulated by posttranslational modifications of histones. In rodents, histone deacetylation produces oligodendrocyte differentiation, whereas acetylation is associated with transcriptional inhibitors of differentiation. In patients with MS, there is a shift toward histone acetylation in the white matter. Thus, hyperacetylation of H3 in the promoter region of inhibitory genes will produce high levels of transcriptional inhibitors of oligodendrocyte differentiation such as *TCF7L2*, *ID2*, and *SOX2* (Table 2) [41].

### 3.3. Nucleosome Positioning and Autoimmune Diseases

Not many studies have been done on how nucleosome positioning causes autoimmune diseases. But in RA, histone variant macroH2A interferes with the binding of transcription factor NF-*κ*B and impedes the action of some proteins that restructure nucleosomes [66]. Also, it has been reported that an SNP in the 17q12-q21 region, which is associated with a high risk of T1D, Crohn's disease, and Primary Biliary Cirrhosis, leads to allele-specific differences in nucleosome distribution [138].

### 3.4. microRNAs and Autoimmune Diseases

#### 3.4.1. Systemic Lupus Erythematosus

Studies have shown that most lupus-related genes contain at least one miRNA target site for more than hundred miRNAs. In SLE, there is evidence of the key role some miRNAs play in its pathogenesis (Table 2). For example, miR-146a is a negative regulator of TLR signaling and its expression is decreased in patients with SLE. Also, this miRNA is a negative regulator of type I IFN pathway and carries out its function by targeting IFN regulatory factor 5 (IRF-5) and STAT-1 (Signal transduction and transcription protein). Therefore, decreased expression of miR-146a in peripheral blood mononuclear cells (PBMCs) may contribute to the enhanced type I IFN production in SLE [42]. Other studies have found that miR-125a was reduced in patients with lupus. This miRNA is expressed in T cells and is a critical transcription factor in the regulation of the chemokine RANTES (Regulated upon Activation, Normal T-cell Expressed, and Secreted). The decreased expression of miR-125a results in the upregulation and elevation of the inflammatory chemokine RANTES in lupus T cells [43].

Additional studies identified the upregulation of miR-21 and miR-148a in CD4+ T cells. One way these miRNAs may act in SLE is through the production of states of hypomethylation of some promoters by repressing DNMTs, which increases the expression of autoimmune-associated methylation-sensitive genes, CD70, and lymphocyte function-associated antigen [44]. Another way would be to inhibit DNMT1 translation via interaction with its 3′-UTR, as is the case with miR-126 [139]. There are other miRNAs that regulate B and T cell immunity such as miR-155. Therefore, the upregulation of miR-155 in lupus B and T lymphocytes may lead to abnormal B-cell activation and abnormal inflammatory T-cell development and cytokine production in patients with lupus [45, 46].

#### 3.4.2. Rheumatoid Arthritis

miRNAs are also critical for RA pathogenesis (Table 2). For example, miR-155 and miR-146 are overexpressed in RASFs. miR-155 expression is enhanced by TNF-*α* and interleukin-1beta (IL-1B), and this enhancement produces an inhibitory effect on metalloproteinase expression in synovial fibroblasts [47]. In addition, miR-146 is an miRNA that is upregulated by proinflammatory cytokines and its function is to downregulate the NF-*κ*B pathway in monocytes. This miRNA has a strong correlation with the levels of TNF-*α* and interleukin-17 (IL-17) [48, 140, 141]. Another miRNA in RA is miR-203, which also causes repression of several metalloproteinase and inhibition of IL-6 [142].

Another miRNA implicated in RA is miR-124, which targets cyclin-dependent kinase 2 (CDK-2). In the basal state, CDK2 represses cell proliferation and arrests the cell cycle at the G1 phase, but in pathologic conditions such as RA, its level decreases. miR-124 also targets monocyte chemoattractant protein 1 (MCP-1), which is responsible for mononuclear phagocytes into the joint. Thus in RA, this miRNA increases cell proliferation and MCP-1 production [49, 143].

#### 3.4.3. Multiple Sclerosis

Currently, many studies have been focusing on miRNAs involved in MS pathogenesis (Table 2). A recent study found that miR-326 plays a critical role in the pathogenesis of MS since it upregulates the Th-17 cell differentiation by targeting Ets-1, which is a negative regulator of Th-17 differentiation. This miRNA was significantly upregulated in patients with relapsing-remitting MS which produced an increase in Th-17 cell numbers and more severe symptoms [50]. Other miRNAs involved in MS are miR-34a and miR-155, which are upregulated in active MS lesions and contribute to MS pathogenesis by targeting CD47. CD47 is a “don't eat me” signal, and macrophages with low levels of this molecule are released from the inhibitory control signal, which causes increased phagocytosis of myelin. Also, miR-155 promotes development of inflammatory Th1 and Th17 cells [51].

In addition, differentially expressed miRNAs such as miR-17-5p, miR-497, miR-193, and miR-126 have been identified in different lymphocyte subsets including CD4+ T cells, CD8+ T cells, B cells, and CD4+ CD25+ Treg cells from patients with MS. Nevertheless, direct involvement and contribution of dysregulated miRNAs in MS has largely remained unknown and needs additional investigation [52]. It is noteworthy that all miRNAs are involved in the pathogenesis of the disease. There are miRNAs that can serve as prognostic markers. For example, the expression of miR-18b and miR-599 is related to relapse and miR-96 is involved in remission [53].

Other miRNAs such as miR-124, which is expressed in microglia but not in peripheral monocytes or macrophages, are brain specific. Their function may be to reduce activation of myelin-specific T cells with a marked suppression of the disease, which would make it a key regulator of microglia quiescence and a good prognostic factor for MS [54].

#### 3.4.4. Type 1 Diabetes

There are few studies related to miRNAs and T1D pathogenesis. But there are some hypotheses that the function of regulatory T cells (Tregs) is influenced by changes in the expression of specific miRNAs (Table 2). In Tregs of diabetic patients, there is an increase in the expression of miRNA-510 and decreased expression of both miRNA-342 and miRNA-191. The exact function of these two is not yet known. There are other studies which demonstrate that miRNAs may be the cause of cytokine-mediated beta-cell cytotoxicity. This cytotoxicity is achieved when IL-1B and TNF-*α* induce the expression of miR-21, miR-34a, and miR-146a in pancreatic islets thus producing beta-cell failure by increasing proinflammatory cytokines [55, 56, 144].

#### 3.4.5. Sjögren's Syndrome (SS)

This syndrome is characterized by the inflammation and dysfunction of salivary and lacrimal glands, which cause dry mouth and eyes. Some miRNAs seem to play an important role in SS: miR-547-3p, miR-168-3p, miR-150, and miR-149 (Table 2). The first two are overexpressed in salivary glands, while the last two are upregulated in salivary glands and lymphocytes. The exact function of each one of these miRNAs has not yet been elucidated, but their overexpression may be the cause of the downregulation of some mRNAs that are important for correct immune function and for the downregulation of proinflammatory cytokines [57, 109].

## 4. Conclusions

Epigenetic research has grown and is now providing new insights into autoimmune diseases. This is possible thanks to advances in technological development, which are enabling epigenomic analysis on a large scale. This improvement in the genetic field has enabled us to find new causes that may explain the etiology of autoimmune diseases and, once again, has shown us that this group of diseases is not caused by a single altered component.

The candidate gene studies have identified a small set of genes that undergo aberrant DNA demethylation and overexpression in SLE and RA, which are the autoimmune diseases that have been the most widely studied in the last few years. This identification of cell-specific targets of epigenetic deregulation in autoimmune rheumatic disorders will provide clinical markers for diagnosis, disease progression, and response to therapies. However, to achieve this, high-throughput approaches are necessary for screening epigenetic alterations in autoimmune diseases related to specific tissue and cell types that are relevant to disease pathogenesis.

Once we have mapped all the altered epigenetic mechanisms that produce each one of the autoimmune diseases, even more research can be done on the therapeutic potential of compounds directed against those epigenetic mechanisms. But to do this, detailed human DNA methylomes, histone modification, and nucleosome positioning maps in healthy and diseased tissues are needed.

## Figures and Tables

**Table 1:** Concordance rate of autoimmune diseases between monozygotic twins.

Autoimmune disease	Concordance rate	References
Systemic lupus erythematosus	11–25%	[7] [8]

Type I diabetes mellitus	13–48%	[9]
		[10]
		[11]
		[12]
		[13]

Rheumatoid arthritis	12–22%	[14]
		[15]
		[16]

Grave's disease	22.2%	[17]

Multiple sclerosis	9–31%	[18]
		[19]
		[20]
		[21]

Celiac disease	75–83%	[22]

**Table 2:** Summary of epigenetic mechanisms involved in autoimmune diseases.

	* DNA methylation*	
Systemic lupus eErithematosus	Global Hypomethylation of promoter region of genes:	References
	ITGAL	[23]
	CD40LG	[24]
	PRF1	[25]
	CD70	[26]
	IFGNR2	[27]
	MMP14	[27]
	LCN2	[27]
	Ribosomal RNA gene promoter (18S and 28S)	[27]
	e1B promoter of CD5 in resting B cells	[28]

Rheumatoid arthritis	Hypomethylation:	
	CpG islands upstream of an L1 open-reading frame	[29]
	IL-6 promoter gene in monocytes	[30]
	Hypermethylation:	
	Promoter of death receptor 3 (DR-3)	[31]

Type 1 diabetes	Global hypermethylation by altered metabolism of homocysteine	[32]

Multiple sclerosis	Hypomethylation of promoter region of peptidyl arginine deiminase type II (PAD2)	[33]

Systemic sclerosis	Hypermethylation of CpG islands in Fli1 promoter	[34]

	* Histone modification*	

Systemic lupus erithematosus	Predisposition to apoptotic nucleosomes	
	H3K4me3	
	H4K8 triacetylation	
	H3K27me3	[35]
	H2BK12ac	
	Global acetylation of histone H3 and H4 in active CD4+ T cells	[36]

Rheumatoid arthritis	HDAC inhibitors:	
	Block induction of MMPs	[37]
	Repress of ADAMTs enzymes	
	Hyperacetylation of histones induces p16 and p21	[38]

Type 1 diabetes	Increase H3K9me2 in lymphocytes genes:	
	CLTA4	
	TGF-B	
	NF-*κ*B	[39]
	p38	
	IL-6	
	Hyperglcemia causes H3K4 and H3K9 methylation	[40]

Multiple sclerosis	Hyperacetylation of H3 promoter region in white matter	[41]

	* miRNAs*	

Systemic lupus Erithematosus	Decreased expression:	
	miR-146a	[42]
	miR-125a	[43]
	Upregulation:	
	miR-21 and miR-148a	[44]
	miR-155	[45]

Rheumatoid arthritis	Overexpression:	
	miR-155	[46]
	miR-203	[47]
	miR-146	[48]
	Decreased expression of miR-124	[49]

Multiple sclerosis	Upregulation:	
	miR-326	[50]
	miR-34a	[51]
	miR-155	[51]
	Expression in Treg cells: miR-17-5p, miR-497, miR-193 and miR-126	[52]
	Disease Relapse: miR-18b and miR-599 Disease Remission: miR-96	[53]
	Brain-specific: miR-124	[54]

Type 1 diabetes	Overexpression of miRNA-510	[55]
	Decreased expression of miRNA-342 and miRNA-191	[55]
	Beta cell failure: miR-21, miR-34a, and miR-146a	[56]

Sjögren's syndrome	Overexpression: miR-547-3p and miR-168-3p	[57]
	Upregulated: miR-150 and miR-149	[57]
